# Analysis of serological markers and synovial fluid cytology in aseptic revision total knee arthroplasty

**DOI:** 10.1007/s00402-026-06536-5

**Published:** 2026-10-20

**Authors:** Farouk Khury, Jack Zhong, Garrett Ruff, Anzar Sarfraz, Laith Bahlouli, Vinay K. Aggarwal, Ran Schwarzkopf

**Affiliations:** 1 The Ruth and Bruce Rappaport Faculty of Medicine, Technion – Israel Institute of Technology, Haifa, Israel; 2 Department of Orthopedic Surgery, New York University Langone Medical Center, New York, USA; 3 Division of Orthopedic Surgery, Rambam Health Care Campus, Haifa, Israel

**Keywords:** Aseptic revision knee, Revision total knee arthroplasty, Aseptic rTKA, Aseptic revision TKA, Aseptic synovial fluid, Aseptic inflammatory markers

## Abstract

**Background and aim:**

Recent studies examining synovial fluid analysis to differentiate causes for aseptic revision total knee arthroplasty (rTKA) were limited to aseptic loosening and instability. This study aims to establish specific serological and synovial profiles for various rTKA etiologies.

**Methods:**

We retrospectively reviewed patients who underwent aseptic rTKA at a single academic institution between 2012 and 2023. Inclusion required complete serum inflammatory markers (erythrocyte sedimentation rate (ESR) and C-reactive protein (CRP)) and synovial fluid differentials within three months pre-operatively. Periprosthetic joint infections were strictly excluded utilizing the 2018 International Consensus Meeting criteria. In total, 156 patients were included and stratified into five aseptic etiologies: aseptic loosening (50.6%), instability (28.2%), arthrofibrosis (16.7%), liner wear (2.6%), and component malposition (1.9%). Serological markers were evaluated utilizing a multivariate general linear model adjusting for patient demographics, while synovial markers were assessed via Welch’s Analysis of Variance (ANOVA) and Games-Howell post-hoc testing.

**Results:**

Multivariate analysis demonstrated that the underlying rTKA indication was not a significant independent predictor of elevated ESR (*P* = 0.661) or CRP (*P* = 0.947). Welch’s ANOVA revealed significant differences in the percentage of synovial monocytes (*P* < 0.001) and lymphocytes (*P* = 0.010), alongside the polymorphonuclear cells/monocyte (*P* = 0.030) and lymphocyte/monocyte (*P* = 0.005) ratios. Games-Howell post-hoc testing demonstrated the tibial liner wear group exhibited significantly higher monocyte percentages, paired with lower lymphocyte percentages and cellular ratios, compared to other aseptic etiologies.

**Conclusions:**

This study revealed that routine systemic inflammatory markers and standard synovial fluid cell counts do not significantly vary among the common etiologies of aseptic rTKA. Nonetheless, tibial polyethylene liner wear appears to elicit an exploratory, monocyte-predominant and lymphocyte-depleted local macrophage response that is absent in other mechanisms of aseptic failure. Further research with larger cohorts is warranted to validate this association and explore its potential as a diagnostic tool.

## Introduction

The incidence of total knee arthroplasty (TKA) continues to rise, leading to a potential increase in associated complications and revision rates [1]. While periprosthetic joint infection (PJI) is a well-recognized and devastating complication, a significant proportion of revision TKA (rTKA) procedures are performed for aseptic indications, including arthrofibrosis, aseptic loosening, component malposition, instability, and liner wear [2, 3]. Diagnostic criteria for PJI have been well standardized through multiple established guidelines, including those from the Musculoskeletal Infection Society (MSIS) in 2011 [4], Infectious Diseases Society of America (IDSA) in 2013 [5], International Consensus Meeting (ICM) in 2013 [6], European Bone and Joint Infection Society (EBJIS) in 2021 [7], and the updated ICM criteria published by Parvizi et al. in 2018 [8]. These criteria incorporate preoperative assessment of serum inflammatory markers – erythrocyte sedimentation rate (ESR) and C-reactive protein (CRP) – and synovial fluid cell counts [8]. However, diagnosing aseptic indications for rTKA remains more challenging, particularly for aseptic loosening and liner wear, as radiographic changes often remain subtle until advanced disease stages result in significant pain or mechanical failure requiring revision [9, 10].

Recent studies have sought to assess synovial fluid biomarkers and serum inflammatory markers across different aseptic revision indications to improve diagnostic accuracy and better understand the inflammatory environment of the joint. However, data on synovial fluid biomarkers in the rTKA population remain limited. One study by Chalmers et al. [11] found that synovial white blood cell (WBC) counts in patients with aseptic loosening did not differ significantly from those in a normal control cohort composed of patients with a prior TKA that was painless, stable on clinical examination, and well-functioning. However, differences in WBC counts were observed between aseptic loosening and other aseptic failure categories. Although fewer studies have been conducted in the rTKA population, research in revision total hip arthroplasty (rTHA) has yielded mixed results. Past studies have reported that synovial WBC counts are greatest in the context of metallosis, polyethylene wear [12], and dislocation [13]. Conversely, a recent paper by Hubert et al. [14] in 2024 reported no difference in synovial cell counts between any aseptic indications. Importantly, these studies have determined that automated cell counts artificially inflate WBC counts in the context of metallosis and liner wear, partly explaining the lack of consensus in the literature.

Given the paucity of data on serum and synovial fluid analyses in aseptic rTKA, our study aimed to assess serum inflammatory markers and synovial fluid cell counts across different aseptic revision indications, including arthrofibrosis, aseptic loosening, component malposition, instability, and liner wear. We hypothesized that etiologies involving particulate debris, such as polyethylene liner wear, would elicit a distinct, localized synovial macrophage response compared to purely mechanical modes of failure.

## Methods

### Study design

**Fig. 1:**
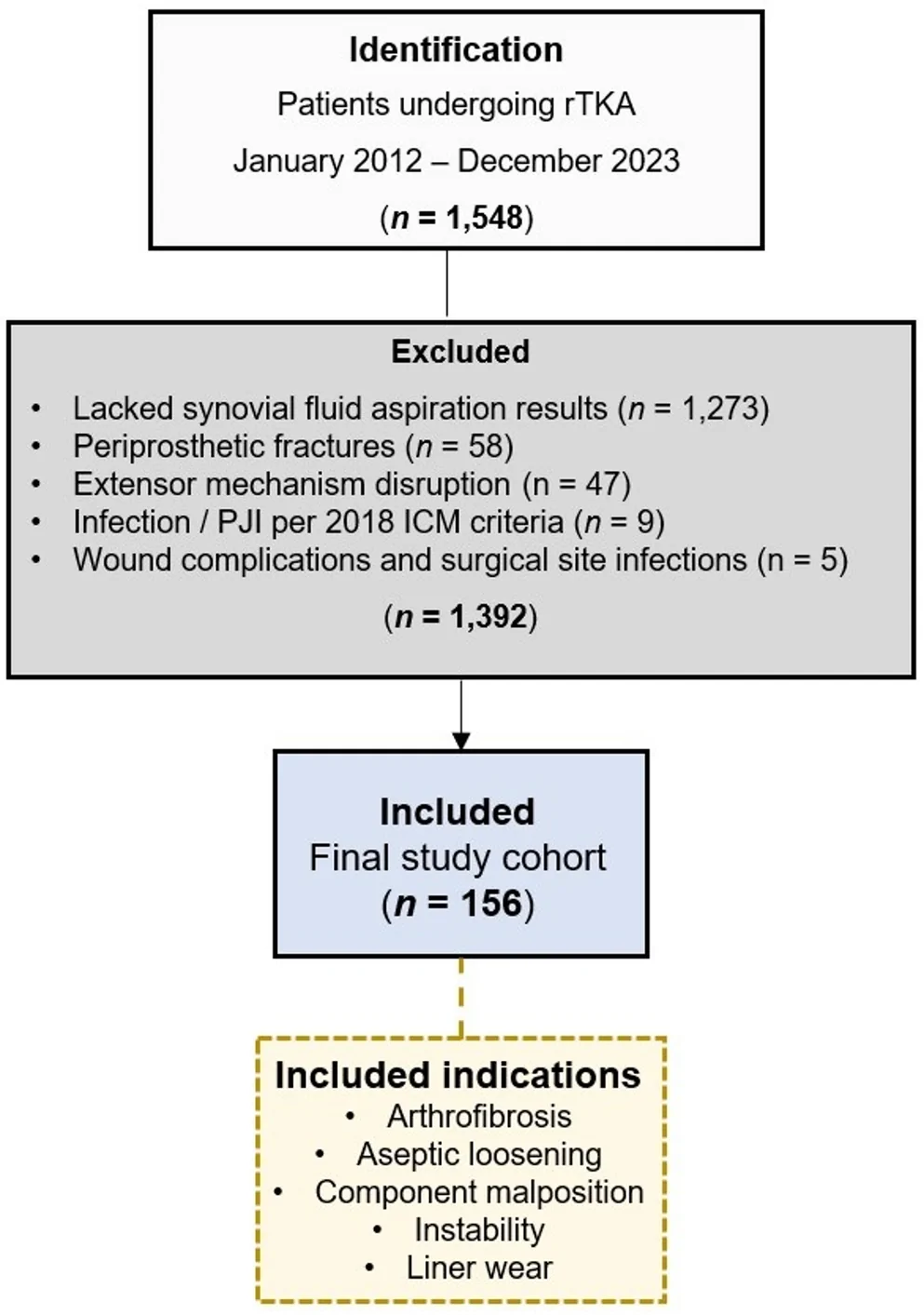
Diagram illustrating the patient selection process. *n*, Number; rTKA, Revision total knee arthroplasty; PJI, Periprosthetic joint infection; ICM, International Consensus Meeting on Musculoskeletal Infection

A retrospective review under an Institutional Review Board-approved research protocol, was conducted of patients who underwent aseptic rTKA between January 1, 2012, and December 31, 2023, at a single, urban, high-volume, academic hospital. Patients were included in the study if they had undergone aseptic rTKA due to arthrofibrosis, aseptic loosening, component malposition, instability, or liner wear, and had negative pre- or intraoperative bacterial cultures. The indication for primary TKA in all patients was primary osteoarthritis. Patients who had positive pre- or intraoperative cultures, had any previous revision due to a PJI, or lacked synovial fluid aspiration results or serum inflammatory markers (ESR and CRP) within three months preoperative to the rTKA were excluded. To ensure a strictly aseptic cohort, PJIs were excluded using the 2018 International Consensus Meeting (ICM) criteria [8], with any patient scoring 6 or higher removed from the analysis. This aseptic classification is further supported by the absence of subsequent PJI-related re-revisions over a mean follow-up of 1.3 ± 1.5 years. Given the consecutive nature of this case series, an a priori sample size calculation was forgone in favor of including every patient who met the inclusion criteria during the study window. Primary query yielded 1,548 patients (Fig. 1). Of these, 1,273 synovial fluid aspirations were either not performed, or the results were unavailable in the electronic medical record, 58 were excluded due to periprosthetic fracture, 47 were excluded due to extensor mechanism disruption, nine patients had synovial fluid results indicative of a PJI, and five patients were excluded due to wound complications and surgical site infection. After applying all inclusion and exclusion criteria, the final analytic cohort consisted of 156 patients suitable for review and statistical analysis.

### Data collection and outcome measures

Baseline demographic and clinical variables—including age, sex, body mass index (BMI), race, smoking history, and American Society of Anesthesiologists (ASA) physical status classification—were extracted from the institutional electronic medical record (EMR; Epic Systems, Verona, WI, USA). The primary etiology for each rTKA was identified through a detailed review of the EMR and operative reports to establish the primary indication for revision. Ambiguous cases involving overlapping failure mechanisms were independently reviewed and resolved by the senior author, allowing for stratification into five distinct aseptic cohorts: arthrofibrosis, aseptic loosening, component malposition, instability, and liner wear. Within this framework, diagnoses of aseptic loosening and component malposition included failure of the femoral component, the tibial component, or both. Additionally, the instability cohort encompassed patients exhibiting extension (varus-valgus) or flexion (anteroposterior) instability, with or without associated instability of the patellar component.

Preoperative laboratory data were captured if performed within three months prior to the revision surgery. In instances where multiple laboratory values were available within this 90-day window, the test performed closest to the surgical date was chosen, which included intraoperative synovial fluid analyses. All preoperative and intraoperative synovial aspirations were performed prior to the administration of therapeutic antibiotics. The specific biomarkers evaluated were serum ESR, serum CRP, and synovial WBC counts with differential analyses. The absolute values and calculated cellular ratios of these markers were subsequently compared across the five aseptic indication cohorts.

### Data analyses

Descriptive statistics for the patient cohort were reported using means and standard deviations for continuous data, while categorical variables were summarized as frequencies and percentages. The statistical methodology was divided according to the specific physiological properties of the evaluated biomarkers. Because systemic inflammatory markers like ESR and CRP can be influenced by non-orthopedic factors, we utilized a multivariate general linear model (GLM) for their analysis. This GLM was designed to control for systemic confounding variables, specifically incorporating patient age, sex, BMI, and smoking history, alongside ASA physical status (analyzed as a continuous covariate to evaluate linear trends). We calculated Beta coefficients (*β*) and adjusted estimated marginal means to establish whether the underlying reason for the rTKA acted as an independent driver of elevated serology when accounting for the patient’s overall health burden. To assess synovial fluid biomarkers—which serve as indicators of the localized joint environment—a Welch’s Analysis of Variance (ANOVA) was performed to compare means across the five aseptic indication cohorts. The assumption of equal variances was tested using Levene’s test, and any necessary post-hoc pairwise comparisons were executed via the Games-Howell test. Statistical significance was established at a *P*-value threshold of < 0.05. For any analyses yielding a significant overall difference, Cohen’s *f* was computed to determine the effect size (ES). The clinical magnitude of Cohen’s *f* was classified using standard thresholds: small (ES ≥ 0.10 to < 0.25), medium (ES ≥ 0.25 to < 0.40), or large (ES ≥ 0.40). A post-hoc power analysis was conducted utilizing G*Power to assess the detectable effect sizes and achieved statistical power given the cohort imbalances. All data modeling and statistical testing were performed in SPSS Statistics (Version 28; IBM Corporation, Armonk, NY, USA).

### Baseline demographics

A total of 156 patients, aged 64.2 years (range, 46 to 87), were included in the study. The mean BMI was 34.1 kg/m^2^ (range, 19.9 to 54.1). The largest racial group was White (39.7%), and most patients were women (76.3%) and never smokers (71.2%). The most common ASA score was II (47.4%). The mean length of follow-up was 1.3 ± 1.5 years. Patients underwent rTKA 6.4 ± 5.8 years after their primary TKA, most commonly due to aseptic loosening (50.6%), instability (28.2%), arthrofibrosis (16.7%), liner wear (2.6%), and component malposition (1.9%) (Table 1). To assess baseline differences between the indication groups, statistical comparisons were performed via Welch’s ANOVA for continuous variables and Fisher’s Exact Test for categorical variables to account for unequal group sizes, which showed no significant differences (*P* > 0.05) (Supplementary Table 1). A post-hoc power analysis revealed that the overall cohort of 156 patients achieved 86% statistical power to detect a medium effect size (Cohen’s *f* = 0.29) at an alpha level of 0.05. However, the severe subgroup imbalance—specifically the liner wear (*n* = 4) and component malposition (*n* = 3) groups—substantially diminished the statistical power of the pairwise comparisons, rendering findings within these specific subgroups exploratory. Furthermore, the pronounced imbalance between groups (e.g., 79 aseptic loosening vs. 4 tibial liner wear cases) significantly limits the statistical validity of the comparative conclusions.

**Table 1:** Baseline characteristics and features of patients who underwent revision total knee arthroplasty

Parameter	Cohort (*n* = 156)
Mean age at surgery [range], (years)	64.2 [46–87]
Mean BMI ± SD [range], (kg/m^2^)	34.1 [19.9–54.1]
Sex, n (%)	
Women	119 (76.3)
Men	37 (23.7)
Race, n (%)	
White	62 (39.7)
Black or African American	54 (34.6)
Hispanic or Latino	3 (1.9)
Asian	3 (1.9)
Other	34 (21.8)
Smoking status, n (%)	
Never	111 (71.2)
Former	38 (24.4)
Current	7 (4.5)
ASA score, n (%)	
I	40 (25.6)
II	74 (47.4)
III	40 (25.6)
IV	2 (1.3)
Mean length of follow-up ± SD, (years)	1.3 ± 1.5
Mean time until revision ± SD, (years)	6.4 ± 5.8
Indication for rTKA, n (%)	
Arthrofibrosis	26 (16.7)
Aseptic loosening	79 (50.6)
Component malposition	3 (1.9)
Instability	44 (28.2)
Liner wear	4 (2.6)

*rTKA* Revision total knee arthroplasty, *SD* Standard deviation, *n* Number, *%* Percentage, *BMI* Body mass index, *ASA* American Society of Anesthesiologists

## Results

### Serological marker analyses

Serological marker analysis demonstrated elevated ESR levels above laboratory reference ranges across all causes of aseptic rTKA (Table 2). Instability had the highest mean ESR (50.3 mm/hr), while component malposition had the lowest (19.7 mm/hr). Univariate analysis demonstrated significant variance in unadjusted mean ESR (*P* = 0.032) across the diagnostic groups, whereas mean CRP showed no significant difference (*P* = 0.541).

**Table 2:** Differences in unadjusted mean serum inflammatory markers across the aseptic revision etiologies using One-way Analysis of Variance

Marker, mean (SD)	Arthrofibrosis (*n* = 26)	Aseptic loosening (*n* = 79)	Component malposition (*n* = 3)	Instability (*n* = 44)	Liner wear (*n* = 4)	Normal range	*P*-value
ESR, (mm/hr)	33.0 (23.6)	37.4 (27.1)	19.7 (12.9)	50.3 (33.3)	23.8 (14.4)	0–10	0.032*
CRP, (mg/L)	8.2 (9.4)	7.3 (9.3)	3.3 (4.1)	13.5 (34.7)	10.8 (5.7)	0–9	0.541

*n* Number, *CRP* C-reactive protein, *ESR* Erythrocyte sedimentation rate, *mm* Millimeter, *hr* Hour, *L* Liter, *mg* Milligram, *SD* Standard deviation, *Significance set as *P*-value below 0.05

To account for systemic confounders, a multivariate analysis was performed adjusting for age, sex, BMI, smoking status, and ASA score (Table 3). For CRP, neither patient demographic factors nor the rTKA indication were significant independent predictors of elevation (*P* = 0.947). Similarly, for ESR, systemic health burden and demographic factors did not independently drive elevation, and the underlying aseptic diagnosis showed no significant independent association (*P* = 0.661). Although parameter estimates indicated an isolated variance between arthrofibrosis and liner wear (*P* = 0.041), the lack of overall categorical significance confirms that systemic inflammatory markers lack the discriminatory power to differentiate between aseptic etiologies.

**Table 3:** Multivariate general linear model analysis of patient demographic parameters associated with systemic inflammatory markers

Demographic parameter	ESR			CRP		
	β	SE	*P*-value	β	SE	*P*-value
ASA	11.1	6.6	0.093	-0.1	5.3	0.985
Indication for rTKA (Ref: liner wear)			0.661†			0.947†
Arthrofibrosis	108.9	52.5	0.041*	10.5	42.9	0.807
Aseptic loosening	81.8	58.3	0.165	8.1	47.7	0.866
Component malposition	56.5	55.9	0.316	18.6	45.8	0.685
Instability	16.3	36.1	0.653	-2.8	29.5	0.925
BMI	-0.2	0.5	0.765	0.1	0.4	0.821
Age at surgery	0.1	0.3	0.789	0.5	0.3	0.152
Smoking status (Ref: current smoker)			0.721†			0.595†
Never smoked	19.2	61.6	0.756	-26.4	50.4	0.602
Former smoker	-36.1	52.4	0.494	-37.8	42.8	0.381
Sex (Ref: women)	45.6	42.8	0.291	-17.2	35.1	0.626

*CRP* C-reactive protein, *ESR* Erythrocyte sedimentation rate, *ASA* American Society of Anesthesiologists, *BMI* Body mass index, *Ref* Reference, *β* Coefficient, *SE* Standard error, *rTKA* Revision total knee arthroplasty

*Significance set as *P*-value below 0.05. † *P*-value represents the omnibus test (tests of between-subjects’ effects) for the categorical variable

### Synovial marker analyses

**Fig. 2:**
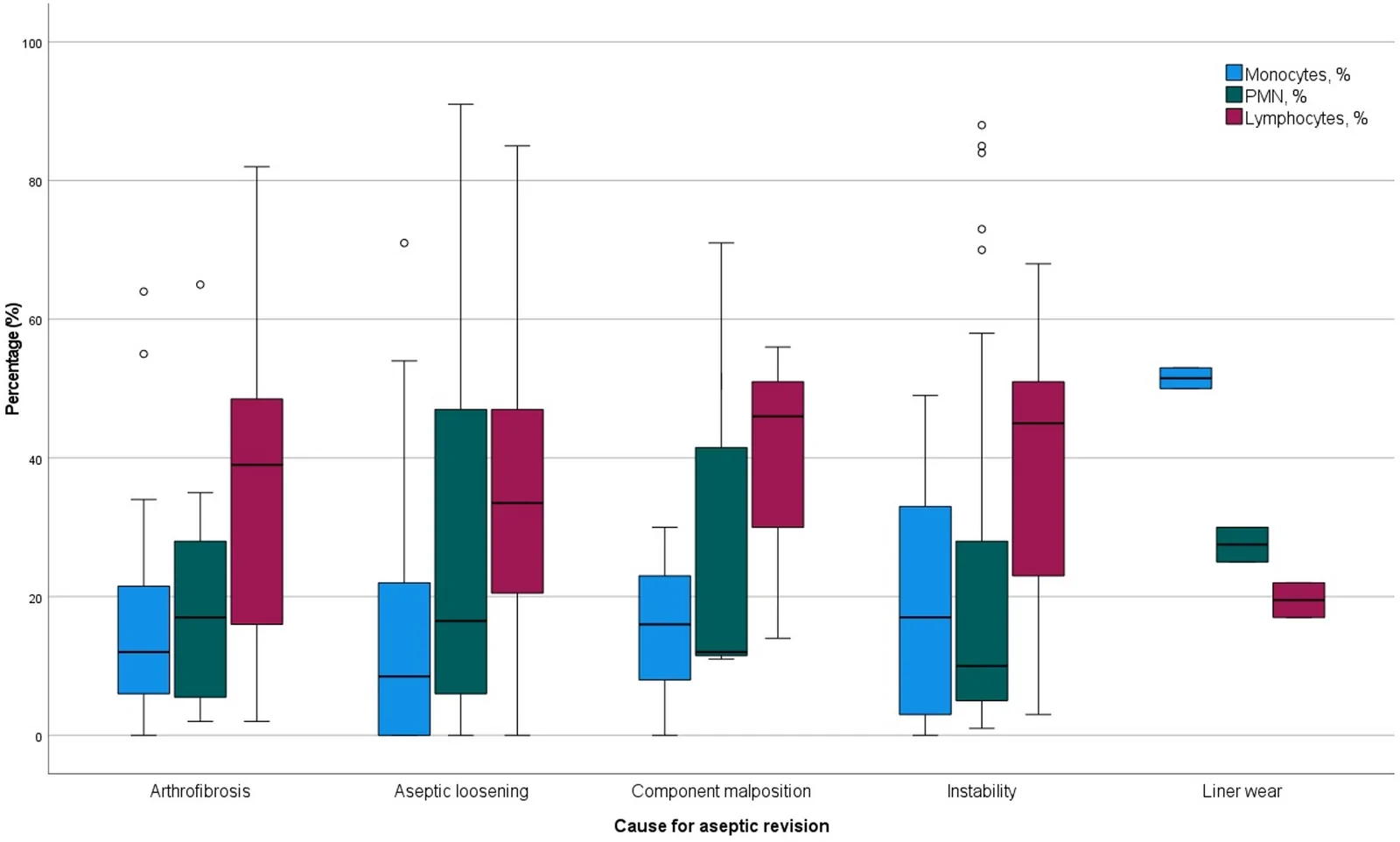
Boxplots that demonstrate the medians, interquartile ranges, and outliers of synovial fluid cytology among the different groups. PMN, polymorphonuclear; %, Percentage

Welch’s ANOVA revealed no significant differences across the aseptic indication groups for synovial WBC counts (*P* = 0.169), RBC counts (*P* = 0.131), proportion of PMN (*P* = 0.570), WBC/RBC ratio (*P* = 0.089), or PMN/lymphocyte ratio (*P* = 0.775) (Fig. 2) (Table 4). However, significant differences were observed in the percentage of synovial monocytes (*P* < 0.001; Cohen’s *f* = 0.29) and lymphocytes (*P* = 0.010; Cohen’s *f* = 0.13), as well as the PMN/monocyte (*P* = 0.030; Cohen’s *f* = 0.12) and lymphocyte/monocyte ratios (*P* = 0.005; Cohen’s *f* = 0.17). Liner wear exhibited the highest monocyte proportion (51.5%). Games-Howell post-hoc testing revealed the monocyte percentage in the liner wear group was significantly higher compared to aseptic loosening (*P* = 0.012), instability (*P* = 0.033), and arthrofibrosis (*P* = 0.029). Furthermore, the liner wear group demonstrated a significantly lower lymphocyte percentage compared to the arthrofibrosis (*P* = 0.047), aseptic loosening (*P* = 0.045), and instability (*P* = 0.018) cohorts, a lower lymphocyte/monocyte ratio compared to the arthrofibrosis (*P* = 0.012) and aseptic loosening (*P* < 0.001) cohorts, and a lower PMN/monocyte ratio compared to the aseptic loosening cohort (*P* = 0.004) (Table 5).

**Table 4:** Synovial marker values and ratios across the aseptic revision groups

Marker or Ratio, mean [SD]	Arthrofibrosis (*n* = 26)	Aseptic loosening (*n* = 79)	Component malposition (*n* = 3)	Instability (*n* = 44)	Liner wear (*n* = 4)	Normal range	*P*-value (ES)
WBC, (10^3^ cells/μL)	0.7 [0.6]	1.2 [1.6]	1.2 [1.0]	0.9 [1.0]	4.7 [6.5]	< 0.2	0.169
RBC, (10^3^ cells/μL)	129.7 [389.7]	90.1 [358.4]	510.3 [651.8]	32.2 [102.3]	9.4 [8.4]	< 1.0	0.131
PMN, (%)	20.8 [16.7]	26.8 [25.2]	31.3 [34.4]	21.6 [25.5]	27.5 [3.5]	< 25	0.570
Lymphocyte, (%)	35.6 [20.7]	35.7 [21.8]	38.7 [21.9]	39.5 [18.0]	19.5 [3.5]	0–10	0.010* (0.13)
Monocyte, (%)	16.8 [17.8]	14.7 [15.6]	15.3 [15.0]	18.3 [15.2]	51.5 [2.1]	0–5	< 0.001* (0.29)
WBC/RBC	0.1 [0.2]	0.2 [0.5]	0.4 [0.1]	0.5 [1.6]	0.6 [0.8]	< 1:1000	0.089
PMN/Lymphocyte	1.2 [1.9]	1.9 [3.3]	1.8 [2.8]	2.1 [6.2]	1.5 [0.4]	< 1:1	0.775
PMN/Monocyte	2.2 [2.9]	3.3 [5.4]	0.5 [0.2]	2.7 [6.0]	0.5 [0.1]	< 1:1	0.030* (0.12)
Lymphocyte/Monocyte	2.9 [2.7]	3.4 [4.3]	2.5 [1.4]	6.4 [15.2]	0.4 [0.1]	1:1–2:1	0.005* (0.17)

Welch’s One-way Analysis of Variance was utilized to compare the means of the five groups. ES, Effect size calculated using Cohen’s *f*, where standard guidelines define the magnitude of the effect as: Small (ES ≥ 0.10 to < 0.25), medium (ES ≥ 0.25 to < 0.40), or large (ES ≥ 0.40)

*n* Number, *WBC* White blood cell count, *RBC* Red blood cell count, *PMN* Polymorphonuclear, *µL* Microliter, *%* Percentage,* SD* Standard deviation

*Significance set as *P*-value below 0.05

**Table 5:** Pairwise comparisons of synovial markers and ratios between aseptic revision groups

Marker	Comparison	Mean difference	*P*-value¶ (Games-Howell)
Lymphocyte (%)	Liner wear vs. arthrofibrosis	-16.1	0.047*
	Liner wear vs. aseptic loosening	-16.2	0.045*
	Liner wear vs. instability	-20.0	0.018*
Monocyte (%)	Liner wear vs. arthrofibrosis	34.6	0.029*
	Liner wear vs. aseptic loosening	36.8	0.012*
	Liner wear vs. instability	33.2	0.033*
PMN/Monocyte	Liner wear vs. aseptic loosening	-2.8	0.004*
Lymphocyte/Monocyte	Liner wear vs. arthrofibrosis	-2.6	0.012*
	Liner wear vs. aseptic loosening	-3.0	< 0.001*

*Significance set at *P*-value < 0.05

*PMN* Polymorphonuclear

¶ All *P*-values are based on the Games-Howell post-hoc test, utilized due to the violation of the homogeneity of variance assumption (Levene’s test *P* < 0.001). Only statistically significant pairwise comparisons (*P* < 0.05) are displayed.

## Discussion

This study addressed the limited data regarding serum and synovial fluid profiles across various etiologies of aseptic rTKAs. In our cohort, aseptic loosening (50.6%) and instability (28.2%) were the most frequent indications for revision. The principal finding of this study is that routine systemic inflammatory markers (ESR and CRP) and standard synovial fluid cell counts (WBC, RBC, and PMN percentages) lack the discriminatory capacity to differentiate between common aseptic rTKA etiologies. However, synovial cytology revealed a significantly elevated proportion of monocytes (*P* < 0.001) and a concurrent decrease in lymphocytes (*P* = 0.010) specifically in patients undergoing revision for polyethylene liner wear, suggesting a potentially distinct localized biological response associated with wear-mediated failure.

Although elevated serum ESR and CRP are paramount in the diagnostic algorithmic workup for PJI [8], the present study demonstrated that these markers hold no discriminatory value in differentiating between distinct aseptic revision etiologies. Because ESR and CRP are highly sensitive but inherently non-specific acute-phase reactants, their systemic elevation is easily confounded by patient-specific comorbidities, rendering them ineffective for isolating localized mechanical modes of failure. This conclusion recontextualizes previous literature. Hardcastle et al. [15] evaluated 228 aseptic rTKAs and found that preoperative abnormalities in ESR or CRP predicted subsequent reoperation for any cause—including both PJI and aseptic loosening. Rather than signaling a specific aseptic mechanism, these markers likely represent a generalized inflammatory state associated with impending global joint failure. Furthermore, while Grzelecki et al. [16] analyzed 1,510 revision arthroplasties to establish strict cutoffs (CRP of 9.6 mg/L; ESR of 28 mm/hr) for differentiating septic from aseptic loosening, our data reveals the danger of applying such thresholds too rigidly within purely aseptic cohorts. However, given the substantially larger cohort analyzed by Grzelecki et al. [16], our unadjusted variations must be interpreted with caution, as elevated baseline markers may reflect broader systemic inflammation, comorbid disease, or occult low-grade infection rather than a specific mechanical diagnosis. In our unadjusted analysis, mean ESR values for instability, aseptic loosening, and arthrofibrosis deceptively exceeded these traditional aseptic thresholds. However, once rigorously adjusted for baseline patient demographics and systemic health burden (ASA score), all significant variance between the diagnostic groups evaporated. Therefore, while obtaining preoperative ESR and CRP remains a non-negotiable step to rule out PJI, clinicians must recognize that systemic serology cannot accurately discriminate between specific mechanisms of aseptic failure, and minor elevations should prompt a search for systemic confounders rather than a specific mechanical diagnosis.

Analysis of the localized joint environment via synovial fluid cytology further challenged the utility of traditional markers while identifying an interesting difference in the synovial cell differential among patients with polyethylene liner wear. Consistent with our serological findings, our Welch’s ANOVA demonstrated no significant differences in synovial WBC or PMN counts across the aseptic indications. This adds critical nuance to prior literature; while Chalmers et al. [11] reported that aseptic loosening yielded significantly higher WBC counts (mean 947 cells/µL) compared to stiffness or component malposition, our raw mean for aseptic loosening (1,204 cells/µL) aligns more closely with Christopher et al. [17], who noted no significant difference when compared to instability. Notably, the existing literature has largely overlooked the specific synovial profile of isolated liner wear. Major previous analyses have either grouped wear-mediated failures into broader categories like aseptic loosening or have focused exclusively on total WBC and PMN percentages to rule out infection, thereby missing subtle shifts in the macrophage population. For instance, while Christopher et al. [17] analyzed synovial monocyte percentages, they restricted their comparison to aseptic loosening versus instability, finding no significant difference. By explicitly stratifying tibial liner wear as an independent diagnostic group and evaluating the full differential, our study identified a uniquely significant elevation in the percentage of synovial monocytes (51.5%) in the tibial liner wear cohort (*P* < 0.001), paired with significantly depressed lymphocyte percentages (*P* = 0.010) and corresponding cellular ratios (PMN/monocyte, *P* = 0.030; lymphocyte/monocyte, *P* = 0.005). This finding bridges a critical gap between preoperative synovial fluid analysis and fundamental histological literature, which has long demonstrated that polyethylene particulate debris actively stimulates a targeted, monocyte- and macrophage-driven inflammatory cascade within the joint space [18, 19]. This aligns with earlier findings by Schwarzkopf et al. [20], who demonstrated a highly monocyte-predominant synovial fluid differential in patients suffering from wear debris synovitis. While standard clinical and radiographic examinations remain the primary methods for determining the mechanism of aseptic failure, the observed synovial profile in the liner wear group provides an interesting area for further study. The finding is biologically plausible given the known monocyte- and macrophage-mediated response to polyethylene debris. However, with only four patients in the liner wear group, these findings should be considered exploratory and hypothesis-generating. This study was not designed to evaluate the diagnostic accuracy of synovial monocyte percentages or establish a threshold for identifying liner wear. Larger studies are needed to determine whether this finding is reproducible and whether it has any diagnostic value in differentiating liner wear from other causes of aseptic revision.

### Limitations

There are several limitations to this study. Because the rate of aseptic revisions overall remains low after primary TKA, the total number of patients included in the study was limited. Additionally, excluding patients without preoperative synovial aspirations resulted in a final cohort representing roughly 10% of the initial query. This introduces selection bias, as aspirated patients inherently represent a cohort with higher diagnostic uncertainty or clinical suspicion for infection. Furthermore, the patient cohort needed to be collected over a course of 12 years. Changes in techniques and technologies in TKA during that time period may affect the presentation of aseptic revision patients from the beginning of data collection to the end. These revision cases and laboratory studies occurred at a single academic institution. An additional limitation stems from the inability to confirm the exact method of synovial fluid analysis—automated counting versus manual microscopy—from the retrospective chart review. Given the established tendency of automated systems to conflate polymeric debris with nucleated cells, the absolute cell counts reported herein may be subject to artifactual elevation, a risk that is especially pertinent to the polyethylene liner wear subgroup. In addition, the lab values are not standardized in terms of the amount of time leading up to the revision TKA, and some patients had intraoperative synovial fluid analyzed. The difference in time point of collection relative to the revision surgery can affect the lab values and their reproducibility in clinical settings. Furthermore, because many primary TKAs were performed at outside institutions prior to the 12-year study window, granular details regarding primary implant design, specific tibial polyethylene formulations, fixation methods, and patient activity levels were inconsistently documented in the electronic medical record. Consequently, it was not possible to report or control for the effect of these specific implant characteristics on the synovial laboratory values. Finally, unmeasured variables such as primary implant constraint levels, variations in tibial polyethylene formulations, and changes in automated laboratory calibration techniques over the 12-year study period serve as temporal confounders. There needs to be a more consistent protocol with regard to the timing and analysis of synovial fluid when aseptic causes of revision are suspected.

### Conclusion

In conclusion, while routine serological markers and standard synovial cell counts are fundamental for excluding periprosthetic joint infection, they offer limited use in differentiating between specific etiologies of aseptic rTKA. We observed a monocyte-predominant and lymphocyte-depleted synovial profile in patients undergoing revision for polyethylene liner wear. However, this finding was based on only four patients and should be considered exploratory and hypothesis-generating. Larger studies are needed to determine whether this finding is reproducible and whether it has any diagnostic value in differentiating liner wear from other causes of aseptic revision.

## Data Availability

The datasets generated and/or analysed during the current study are available from the corresponding author on reasonable request.
